# Position Paper on the Management of Sickle Cell Disease in Saudi Arabia: Understanding Disease Landscape, Identifying Challenges, and Exploring Opportunities in Transfusion Therapies

**DOI:** 10.3390/jcm14103494

**Published:** 2025-05-16

**Authors:** Hind AlHumaidan, Abdullah Al Zayed, Ahmed Al Bahrani, Ammar Hasan Alsughayir, Kefah Baqir Algadeeb, Nour Al Mozain, Ohoud Kashari, Tahani Ali Mohamed Bin Ali, Hafiz Malhan

**Affiliations:** 1 Department of Pathology, King Abdullah Bin Abdulaziz University Hospital, Princess Nora University, Riyadh 13412, Saudi Arabia; 2 Department of Hematology, Qatif Central Hospital QCH, Qatif 32654, Saudi Arabia; abalzayed@moh.gov.sa; 3 Department of Blood Bank and Transfusion Medicine, King Fahad Specialist Hospital, Dammam 32253, Saudi Arabia; atalbahrani@hotmail.com; 4 Department of Blood Bank and Transfusion Services, King Fahad Medical City, Riyadh 12231, Saudi Arabia; aalsughayir@kfmc.med.sa; 5 Department of Hematology, King Fahad Hospital-Hofuf, Al Hofuf 36441, Saudi Arabia; kefahbaqir@gmail.com; 6 Department of Pathology and Laboratory Medicine, King Faisal Specialist Hospital, Riyadh 11564, Saudi Arabia; nalmozain@kfshrc.edu.sa; 7 Department of Pediatric Hematology, Jeddah Second Health Cluster, Jeddah 23816, Saudi Arabia; dr.o.kashari@gmail.com; 8 Department of Pediatrics Hematology Oncology, Prince Sultan Military Medical City, Riyadh 11159, Saudi Arabia; tbinali@psmmc.med.sa; 9 Department of Hematology, Prince Mohammed Bin Nasir Hospital, Jazan 82843, Saudi Arabia; dr-hafiz@hotmail.com

**Keywords:** sickle cell disease, Saudi Arabia, clinical practice, position paper, aRBCX, red blood cell exchange, transfusion therapy, expert opinion

## Abstract

Sickle cell disease (SCD) is a common genetic disorder affecting up to 2.6% of the population in Saudi Arabia. SCD results in severe disability, reduced quality of life, extensive use of medical resources, increased economic burden, and a high likelihood of increased mortality. Red blood cell transfusion remains a cornerstone in the management of SCD complications. This position paper highlights the current state of SCD management within the Kingdom of Saudi Arabia. Despite the advantages of automated red blood cell exchange (aRBCX) and guideline recommendations, its use remains limited. In practice, aRBCX is used for a variety of indications, including acute management and prophylaxis of stroke, systemic fat embolism, severe forms of acute chest syndrome, preoperative management, hematopoietic stem cell transplantation, hepatic crisis, and priapism. However, aRBCX is underutilized in pregnancy. Common gaps identified by the advisory panel include the absence of standardized national guidelines, limited access to aRBCX, issues with vascular access, lack of equipment, and insufficient staff training. Another limitation to the use of aRBCX is the higher blood requirements compared to other blood transfusion modalities. These factors contribute to geographical disparities in the management of SCD and suboptimal patient outcomes. To address these issues, the advisory panel recommended developing and implementing evidence-based national guidelines, expanding access to aRBCX, enhancing health staff education and training, and establishing a robust national SCD registry. By prioritizing these recommendations, we can help streamline SCD care, reduce practice variation, and nationalize sickle cell disease management in Saudi Arabia to improve patient care.

## 1. Background

Sickle cell disease (SCD) is one of the most common inherited single-gene blood disorders, affecting nearly 8 million people worldwide [1]. SCD occurs due to mutations in the β-globin gene, which lead to the production of sickle hemoglobin (HbS), ultimately resulting in the sickling of red blood cells [1,2]. The accumulation of the malformed red blood cells occludes capillaries and prevents tissue oxygen delivery, leading to acute and chronic pain; severe anemia; progressive multiorgan damage; and an increased risk of infection, acute chest syndrome (ACS), stroke, and other cardiovascular diseases [2,3,4]. SCD results in severe disability, reduced quality of life, extensive use of medical resources, increased economic burden, and a high likelihood of increased mortality [1,2]. Results from the International Sickle Cell World Assessment Survey (SWAY), which aimed to provide insights into the patient-reported impact of SCD on their quality of life, revealed that SCD significantly and negatively impacts patients’ quality of life, emotional well-being, education, and employment. Additionally, patients reported experiencing a high number of vaso-occlusive crises (VOCs) and other debilitating symptoms such as fatigue, depression, and anxiety [5].

In Saudi Arabia, the Saudi Premarital Screening Program estimates that 4.2% of adults are carriers for the sickle cell trait, and 0.26% of the population is diagnosed with SCD [6,7]. This makes it one of the highest prevalence rates worldwide [6,7]. This could be attributed to the high prevalence of consanguineous marriages, among other cultural factors [8,9]. SCD is more prevalent in the eastern and southwestern regions compared to other parts of the kingdom [6,10]. A cross-sectional study conducted in 2020 aimed to identify barriers to health services provided to adult SCD patients in the Eastern province. Almost half of the patients reported feeling stigmatized due to their recurring pain episodes, and a third of patients reported inadequacy in the health service provided, along with poor communication from the healthcare staff, all leading to reduced QoL [11]. This highlights the necessity for enhanced healthcare provision through nationwide implementation of patient counseling and empowerment strategies.

Several curative and disease-modifying treatments are available for SCD, including hydroxyurea, red blood cell transfusion, and stem cell transplantation [2]. Red blood cell transfusion remains a cornerstone in the management of SCD, which can alleviate or prevent many complications [12]. Red cell transfusion enhances oxygen delivery to the tissues and reduces the number of sickled red blood cells in the circulation, making it a key intervention in both the chronic management of SCD as well as in its acute complications [13]. Simple transfusion (ST), manual red blood cell exchange (mRBCX), and automated red blood cell exchange (aRBCX) are the currently available transfusion modalities (Table 1) [14]. Accumulating evidence suggests that aRBCX is a rapid, effective, and well-tolerated procedure for patients with SCD, providing good control of sickle hemoglobin percentage without increasing viscosity. aRBCX is also associated with a decreased rate of iron loading and does not result in higher rates of alloimmunization compared to other transfusion techniques, despite the use of additional blood bags [15,16,17].

Consequently, the National Institute of Clinical Excellence (NICE) and the American Society of Hematology (ASH) have recommended that aRBCX should be considered for patients who require regular transfusions [18]. However, aRBCX use remains limited due to the higher blood requirements, venous access, the need for specialized training, and the cost of the device [16].

Regarding the cost of the procedure, aRBCX appears to be most expensive in the short term, primarily due to the high number of blood bags required. However, this transfusion technique has been reported to be cost-saving in the medium to long-term [19,20,21]. These limitations are also important to take into consideration if on-call aRBCX procedures are performed [22].

Additionally, despite the high prevalence of SCD in Saudi Arabia, which poses significant challenges to the healthcare system, there is a lack of national guidelines and country-specific plans for effective management strategies, leading to huge variation in practice [23,24,25]. This calls for combined efforts to nationalize SCD management in Saudi Arabia to streamline practice and improve patient care through leveraging current effective therapies.

To address these challenges, Terumo Blood and Cell Technologies (Terumo BCT, Zaventem, Belgium) Medical Affairs EMEA department organized an advisory board meeting to evaluate the local landscape of SCD and current treatment modalities, particularly aRBCX, and explore solutions to bridge the gaps in clinical practice for the management of sickle cell disease patients in Saudi Arabia.

## 2. Methodology

Terumo BCT Medical Affairs department organized an advisory board meeting in March 2024 in Saudi Arabia. The meeting brought together experts in the field to discuss local clinical practices for managing SCD and highlight key areas for improvement. Nine experts convened with Terumo BCT Medical Affairs representatives. The panel was carefully selected to provide an accurate representation of the situation in Saudi Arabia and included consultant hematologists and transfusion medicine specialists.

### 2.1. Objectives

The purpose of this meeting was to collect insights on the clinical experience of these experts regarding the prophylactic and therapeutic options currently available for patients with SCD in Saudi Arabia, with an emphasis on aRBCX and other transfusion therapies. Different points of discussion were raised, according to the following objectives:

- Understand SCD landscape and disease burden in Saudi Arabia.
- Discuss transfusion indications in the management of acute and chronic SCD complications and their application in clinical practice.
- Gain insights regarding the availability of national guidelines and their significance for a population severely affected by SCD, like Saudi Arabia.
- Understand how international guidelines are applied in local practice.
- Discuss the future of sickle cell disease management in the country.
- Discuss current limitations and possible solutions.

### 2.2. Advisory Board Meeting

The advisory board meeting lasted for 4 h and utilized open discussions as a tool to gather in-depth insights on a variety of issues related to SCD management. A brief introduction was made on the current transfusion modalities and the differences between them, along with a summary of the current guidelines on the use of red blood cell exchange. Following this introduction, the advisory board discussed a number of topics, including SCD burden in Saudi Arabia, aRBCX indications, use of aRBCX in the management of acute and chronic complications, use of aRBCX in pregnancy, and current challenges and recommendations. It is worth noting that all relevant materials and guidelines were shared with the expert panel before the meeting.

Prior to the commencement of the advisory board meeting, all participants were informed of the nature and purpose of the meeting and gave consent for the use of their opinions in the development of this position paper.

### 2.3. Position Paper Development and Approval

This position paper was drafted, considering supporting evidence, based on the insights and recommendations shared by the expert panel during the advisory board meeting. The final draft of the manuscript was shared with the experts over email to acquire their approval.

## 3. SCD Burden in Saudi Arabia

Genetic disorders, such as sickle cell disease and β-thalassemia, are quite prevalent in Saudi Arabia. This could be attributed to the high prevalence of consanguineous marriages, exceeding 55%, and acting as a significant risk factor for these disorders [8,9,26,27]. Other cultural factors contribute to the dilemma, including the large family size and the high paternal and maternal ages [6,9]. In Saudi Arabia, the government has acknowledged these factors and has put in place various awareness and surveillance programs to prevent high-risk marriages and decrease the incidence of genetic diseases. The most effective initiative is the Premarital Screening and Genetic Counseling Program, which has led to a 60% reduction in the number of at-risk couples and a five-fold increase in the number of voluntary cancellations of marriage proposals among at-risk couples [6].

In Saudi Arabia, the estimated prevalence of the sickle cell gene in the adult population is 4.2% for the sickle cell trait and 0.26% for SCD, while 24 out of 1000 children and adolescents are affected by the disease [6,10,28]. SCD in Saudi Arabia displays clinical and hematological variability, with two major phenotypes: a mild phenotype known as the Arab Indian type and a severe phenotype referred to as the African type (Benin haplotype) [28]. Patients in the eastern province have the Arab Indian (mild) phenotype, whereas the Benin haplotype is more frequent in the southern region [29]. While several studies suggested that the Arab Indian phenotype is benign in children due to their high fetal hemoglobin (HbF), the phenotype of adult patients with Arab Indian SCD is not benign despite their relatively high HbF level. This is probably due to the continued decline in HbF levels in adults [29]. Although the disease in the Eastern province has many mild features, splenic complications, such as sequestration crises, chronic hypersplenism, splenic infarction, and splenectomy, are more common because of the late persistent splenomegaly; in addition, complications such as pain crises and vasculopathy occur at a later age [28]. A hospital-based study that aimed to assess SCD complications in adults with the Arab Indian haplotype in the Eastern region showed that almost 96% of patients had a history of painful crises, and almost 46% had at least one episode of ACS. Other complications were reported, including symptomatic osteonecrosis, priapism, and overt stroke. The majority of patients had persistent splenomegaly and gallstones [29]. Previous studies have also demonstrated that the differences between the Arab Indian and African haplotypes are almost negligible except for the absence of leg ulcers, reduced prevalence of overt stroke, and higher rate of persistent splenomegaly in the Arab Indian haplotype [29].

During the discussion, the advisory panel agreed that Saudi Arabia has one of the highest prevalences of sickle cell disease in the world, with up to 2.6% of the population affected. A high prevalence has also been reported in sub-Saharan Africa, the Mediterranean, the Middle East, and India, with approximately 90% of the world’s sickle cell disease population living in Nigeria, India, and the Democratic Republic of Congo, where the disease affects up to 2% of the population [30,31]. In the US, Sickle cell disease affects more than 100,000 people [32]. The severity of SCD in Saudi Arabia varies widely, likely due to existing haplotype variations. Generally, it is believed that sickle cell disease patients with the Arab-Indian haplotype have a generally milder disease and fewer complications compared to the African haplotype. However, in adulthood, both phenotypes can present with complications, with the Arab-Indian haplotype presenting with more ACS, systemic fat embolism, and avascular necrosis (AVN) compared to the African haplotype, where stroke is the most prevalent complication.

## 4. Local Disease Landscape

SCD is widespread in southern and eastern Saudi Arabia. In the eastern province, the prevalence is 145 cases per 10,000 population, significantly higher than in the southern region (24 cases per 10,000 population), western region (12 cases per 10,000 population), and central region (6 cases per 10,000 population) [10]. Even with the higher rate of immigration and advanced services in the central region, the majority of patients with sickle cell disease still lie in the southwestern and eastern regions, especially acute cases where immediate treatment is often required. Importantly, the advisory panel highlighted that they rarely encounter young patients with sickle cell disease as patients are referred to them in later disease stages after developing complications.

## 5. Transfusion Therapies for SCD

Red blood cell transfusion remains an invaluable treatment modality for the management of acute and chronic SCD complications, but it does not come without complications [12]. The main complications associated with blood cell transfusion include alloimmunization, iron overload, hyperhemolysis, hemolytic transfusion reactions, and transfusion-related infections [12,33,34]. Simple transfusion (ST), manual red blood cell exchange (mRBCX), and automated red blood cell exchange (aRBCX) are the currently available transfusion modalities. Simple transfusion is an easy procedure that does not require expertise or equipment; however, it is associated with iron overload, less reduction in HbS levels, and an increase in blood viscosity. Red blood cell exchange, which includes mRBCX and aRBCX, allows the patient’s blood to be removed and replaced. mRBCX is also laborious, less effective in reducing the HbS level, and can still lead to a degree of iron overload [16]. aRBCX is performed through an apheresis system where the patient’s own red cells are simultaneously removed and replaced with donor red cells. This process requires specialist staff and equipment but can rapidly reduce the HbS level, avoiding iron loading more easily. Hence, it is the recommended mode of delivering chronic transfusions for patients with SCD [14]. A retrospective observational study aimed to compare the safety and efficacy of chronic automated red blood cell exchange and manual exchange transfusion in patients with sickle cell disease in Saudi Arabia. In this study, aRBCX proved to be more effective in regard to HbS reduction, fewer hospital visits, and better disease control when compared to mRBCX, with no increased alloimmunization risk [15].

In practice, there is a tendency to delay the initiation of blood transfusions until necessary to avoid potential complications. The advisory panel also highlighted that even with the availability of international guidelines for the management of sickle cell disease, it is paramount that treating physicians individualize treatment based on patient factors such as age, values, and preferences and also take into account other logistic constraints, such as access to treatment [14].

## 6. aRBCX

### 6.1. Guidelines

Current guidelines have discussed different transfusion modalities for patients with SCD and have developed several recommendations to support their management. ASH recommends the use of aRBCX over mRBCX and ST in patients who require chronic transfusion [14]. However, certain factors must be considered, including patient age, patient preferences, feasibility, availability of blood, as well as the clinical indication for chronic transfusion. ASH also recommends the use of aRBCX along with mRBCX in patients with severe acute chest syndrome. For moderate ACS, ASH guidelines suggest the use of any of the available transfusion modalities and did not support the use of one over the other. Red cell exchange is also recommended for pregnant patients with SCD either as prophylaxis at regular intervals or to be carried out when indicated. Similarly, the NICE guidelines have also endorsed the use of aRBCX for patients with sickle cell disease who require regular transfusions [18]. The American Society for Apheresis (ASFA) guidelines also recommend RBCX (mRBCX and aRBCX) for acute complications, namely acute stroke and acute chest syndrome, and chronic complications, including stroke prophylaxis, priapism, recurrent VOCs, pregnancy, and pre-operative management [35].

### 6.2. aRBCX Indications and Utilization in Clinical Practice

The advisory board meeting discussed the current indications for aRBCX considering the international guidelines and local clinical practice. Due to the absence of national guidelines for the management of sickle cell disease in Saudi Arabia, the ASFA, ASH, and NICE guideline recommendations were discussed. The advisory panel noted that most hematologists are following the ASFA and ASH guidelines. However, there still are variations in practice due to limited access to the treatment in some centers. This can be explained notably by a lack of knowledge, lack of resources, vascular access issues, and blood availability.

Generally, aRBCX is used for many indications in local practice, including acute management and prophylaxis of stroke, systemic fat embolism, severe forms of ACS, preoperative management, hematopoietic stem cell transplantation (HSCT), hepatic crisis, and priapism. aRBCX is not routinely done for vaso-occlusive pain crises but may be considered for severe recurrent cases. According to the advisory panel, aRBCX is underutilized in pregnancy despite the ASFA recommendations and recent supporting evidence [35,36].

#### 6.2.1. Acute and Chronic Management of Stroke

Stroke is one of the serious complications associated with SCD. The role of transfusion therapy in stroke prophylaxis and for the management of acute ischemic stroke is well-established in children with SCD and is strongly recommended [12]. There is limited data on stroke prevention in adults with SCD. However, current guidelines recommend chronic transfusion therapy for adults who have suffered an ischemic stroke due to SCD. In the acute setting, red blood cell exchange is often used because it can promptly reduce HbS to <30% while maintaining a stable blood volume. A retrospective study demonstrated that using exchange transfusion at the onset of a stroke was linked to a reduced risk of subsequent stroke in comparison to simple transfusion [37]. For patients with SCD who experience an overt stroke or have abnormal transcranial Doppler ultrasound (TCD), ASH recommends HLA-matched related stem cell transplantation over a chronic exchange program [38].

#### 6.2.2. Acute Chest Syndrome (ACS)

There is a clear variation in practice when it comes to the management of ACS for patients with SCD. While aRBCX is sometimes used for patients with severe ACS associated with shortness of breath or multiorgan failure, some physicians refrain from using automated transfusion and rely on other treatment modalities depending on the severity of the presentation. Some physicians can opt for a simple transfusion and, after failure, move to automated transfusion. Even though the currently available evidence for RBCX in the management of ACS is limited, several guidelines recommend the use of red blood exchange for patients with severe ACS in the acute setting [12,35].

#### 6.2.3. Pregnancy

In Saudi Arabia, the use of aRBCX for pregnant patients with SCD is not a common practice. However, patients who become pregnant may require transfusion therapy. ASFA lists pregnancy in SCD patients as one of the indications where transfusion therapy might be recommended (category II) [35]. Additionally, the recent findings of an international Delphi panel recommend that patients with a history of poor maternal outcomes during previous pregnancies, including serious complications, frequent hospitalizations, or severe pain, should consider receiving prophylactic transfusion therapy. It is important to note that the only contraindication to the use of aRBCX during pregnancy is if the patient has experienced serious transfusion complications or reactions in the past or has multiple alloantibodies [36].

In local practice, aRBCX is only carried out for pregnant women classified as high-risk patients or who have underlying comorbidities that necessitate transfusion therapy. Despite the risks of congenital abnormalities and infertility in children later in life [39], small doses of hydroxyurea can be used for pregnant women with SCD in some centers. This practice is supported by limited data indicating that exposure of the fetus to low doses of hydroxyurea does not result in teratogenic changes [40].

#### 6.2.4. Priapism

Priapism is a painful, major complication of SCD among males that can lead to erectile dysfunction [41]. In cases of acute priapism, aRBCX is sometimes used, but limited evidence supports its efficacy [41,42]. There is also a lack of evidence on the primary and secondary prevention of priapism [41].

In local practice, advisors reported the common use of aRBCx for the prophylactic and acute management of priapism. Nevertheless, current guidelines do not include priapism as one of the strong indications for aRBCx. The ASFA guidelines have highlighted that RBC transfusion may be considered pre-operatively if surgical intervention is needed. They also mentioned that limited evidence supports RBC exchange as a treatment modality to resolve priapism within one to two days [43].

## 7. Advantages and Limitations to the Use of aRBCX in Clinical Practice

aRBCX stands as a promising transfusion modality for patients with SCD owing to its effectiveness and safety [17]. Indeed, it is a rapid process that simultaneously removes the patient’s RBCs and replaces them while maintaining isovolemia. This technique avoids the increase in blood viscosity and associated risks that are often seen with other transfusion modalities. aRBCX is also associated with reduced iron overload and faster and greater HbS reduction [15,16,17].

However, despite the advantages of aRBCX and its many indications, it also faces some disadvantages. Common limitations to its use include the higher number of blood units needed for automated exchange, accounting for almost 25% higher than mRBCX. However, it is important to note that aRBCX allows for reducing the frequency of transfusion treatment, diminishing the number of blood bags requested in the long term. The panel mentioned some additional gaps regarding access to aRBCx treatment, among them, nurses’ training and lack of equipment like MRI machines to assess patients with SCD for disease-related complications [44] and ultrasound-guided venous access.

These gaps are considered universal and have been reported before [13,16]. Limited availability, significant staff training, and high requirements for central access and advanced devices are common challenges to the utilization of RBCs [13,16]. Despite these requirements, aRBCx remains a cost-effective treatment option and the recommended transfusion modality for chronic red blood cell transfusion [18].

## 8. Plasma Exchange in SCD Management

The advisory panel deemed plasma exchange to be a promising option for SCD patients due to improvements in clinical status, lactate dehydrogenase (LDH) levels, and platelet counts. In clinical practice, plasma exchange is used for severely ill patients who do not improve after aRBCX. Despite the lack of available evidence, plasma exchange is becoming a standard of care for patients who do not respond to RBC exchange in some centers in Saudi Arabia.

Some studies and case reports have reported similar practices involving therapeutic plasma exchange for patients with acute complications of SCD who do not improve after aRBCX. These data demonstrated that plasma exchange led to rapid pain resolution and favorable clinical outcomes while being well tolerated [45,46]. However, randomized controlled trials are still lacking in the literature.

## 9. Gaps in Clinical Practice

Despite the high burden of SCD in Saudi Arabia, the experts highlighted that there is a lack of nationwide prospective studies to estimate the prevalence, molecular, and clinical epidemiology of SCD in different regions of the Kingdom [26,28]. Additionally, there is no national registry for patients with SCD, which could further aid in the true estimation of this common disorder. The advisory panel concurred that no national guidelines are in place for the management of SCD in Saudi Arabia, resulting in significant variation in practice among different centers. Consequently, hematologists and transfusion consultants mainly follow the ASFA and ASH guidelines for managing patients with SCD. The panel mentioned some additional gaps in regard to access to aRBCx treatment, nurses’ training, lack of equipment like MRI machines and ultrasound-guided venous access, and blood bank logistics (e.g., providing the necessary blood units for aRBCX when needed).

## 10. Future Directions

The advisory panel suggested several recommendations for advancing the management of sickle cell disease in Saudi Arabia, as summarized in Table 2**.** These include developing and implementing evidence-based national guidelines, expanding access to aRBCX, enhancing health staff education and training, and establishing a robust national SCD registry. By prioritizing these recommendations, we aim to streamline SCD care, reduce practice variation, and nationalize sickle cell disease management in Saudi Arabia to improve patient care.

## 11. Conclusions

This advisory board meeting allowed the expert panel to comprehensively evaluate the SCD landscape in Saudi Arabia, shedding light on the current disease burden, gaps in clinical practice, and transfusion modalities with a focus on aRBCX. The advisory panel acknowledged the high disease burden in the Kingdom and the need to develop strategies for effective disease control and management. Major gaps in the management of SCD in the Kingdom are summarized as a lack of national guidelines, nationwide studies, a national registry, education and awareness, and centers of excellence for the management of sickle cell disease.

Regarding transfusion therapy, the panel recognized the advantages and indications of aRBCX in the management of acute and chronic SCD complications. The advisory panel emphasized the many limitations that hinder the optimal utilization of aRBCX, including limited availability, significant staff training, and high requirements for central access and advanced devices. Lastly, the panel provided recommendations to advance the management of SCD in Saudi Arabia and improve patient care.

## Figures and Tables

**Table 1:** Comparison of current transfusion modalities.

	Simple Transfusion (ST)	Manual Red Blood Cell Exchange (mRBCX)	Automated Red Blood Cell Exchange (aRBCX)
Definition	Direct transfusion of healthy donor RBCs without removing patient’s RBCs	Manual removal of patient’s RBCs via phlebotomy and replacement with healthy donor RBCs	Simultaneous automated removal and replacement of RBCs using apheresis machine
Time Required	Time consuming	Time consuming and labor intensive	Less time consuming compared to other transfusion modalities
Hyperviscosity	Risk of hyperviscosity with high Hb targets	Less risk of hyperviscosity	Less risk of hyperviscosity
Iron Overload	High, typically requires chelation	Low, sometimes requires chelation	Lowest, does not require chelation
Efficacy in HbS Reduction	Limited, as sickled RBCs remain in circulation	Moderate, but operator dependent	Highest, with precise HbS reduction and hematocrit control
Venous access	Peripheral access is used	Intermediate requirement for central access	High requirement for central access
Intervals between Procedures	Short intervals between procedures	Intermediate intervals between procedures	Long intervals between procedures
Cost and Availability	Low cost, widely available	Moderate cost, requires trained staff	Higher cost in the short-term, requires specialized machines and trained personnel—cost-saving in the medium to long-term

**Table 2:** The advisory panel recommendations to advance the management of sickle cell disease in Saudi Arabia.

Recommendations
The development of a national unified guideline for the management of sickle cell disease in Saudi Arabia to streamline the practice and improve patient care.
2. The establishment of the Saudi Apheresis Society, which is a requirement to create a national registry for apheresis.
3. Emphasizing partnerships, such as with international SCD organizations or research institutions, could strengthen Saudi Arabia’s national approach to SCD management and foster resource sharing or guideline development.
4. Increasing the competency of the nursing staff through tailored educational and training programs.
5. Increasing the availability of ultrasound-guided venous access machines in healthcare facilities.
6. Empowering patients through increasing awareness, education, and the development of a patient society for SCD patients.
7. Solving the venous access dilemma through nurse training, appropriate equipment, and managing chronic patients in an outpatient setting.
8. Establishing a center of excellence for the management of sickle cell disease patients.
9. Local data generation and research support.

## References

[1] Thomson A.M., McHugh T.A., Oron A.P., Teply C., Lonberg N., Tella V.V., Wilner L.B., Fuller K., Hagins H., Aboagye R.G. (2023). Global, regional, and national prevalence and mortality burden of sickle cell disease, 2000–2021: A systematic analysis from the Global Burden of Disease Study 2021. Lancet Haematol..

[2] Brandow A.M., Liem R.I. (2022). Advances in the diagnosis and treatment of sickle cell disease. J. Hematol. Oncol..

[3] Rees D.C., Williams T.N., Gladwin M.T. (2010). Sickle-cell disease. Lancet.

[4] Elendu C., Amaechi D.C., Alakwe-Ojimba C.E., Elendu T.C., Elendu R.C., Ayabazu C.P., Aina T.O., Aborisade O., Adenikinju J.S. (2023). Understanding Sickle cell disease: Causes, symptoms, and treatment options. Medicine.

[5] Osunkwo I., Andemariam B., Minniti C.P., Inusa B.P., El Rassi F., Francis-Gibson B., Nero A., Trimnell C., Abboud M.R., Arlet J.B. (2021). Impact of sickle cell disease on patients’ daily lives, symptoms reported, and disease management strategies: Results from the international Sickle Cell World Assessment Survey (SWAY). Am. J. Hematol..

[6] Memish Z.A., Saeedi M.Y. (2011). Six-year outcome of the national premarital screening and genetic counseling program for sickle cell disease and β-thalassemia in Saudi Arabia. Ann. Saudi Med..

[7] Nasserullah Z., Al Jame A., Abu Srair H., Al Qatari G., Al Naim S., Al Aqib A., Mokhtar M. (1998). Neonatal screening for sickle cell disease, glucose-6-phosphate dehydrogenase deficiency and a-thalassemia in Qatif and Al Hasa. Ann. Saudi Med..

[8] El-Hazmi M.A.F., Al-Swailem A.R., Warsy A.S., Al-Swailem A.M., Sulaimani R., Al-Meshari A.A. (1995). Consanguinity among the Saudi Arabian population. J. Med. Genet..

[9] Al-Gazali L., Hamamy H., Al-Arrayad S. (2006). Genetic disorders in the Arab world. BMJ.

[10] Al-Qurashi M.M., El-Mouzan M.I., Al-Herbish A.S., Al-Salloum A.A., Al-Omar A.A. (2008). The prevalence of sickle cell disease in Saudi children and adolescents. A community-based survey. Saudi Med. J..

[11] Al Sayigh N.A., Shafey M.M., Alghamdi A.A., Alyousif G.F., Hamza F.A., Alsalman Z.H. (2023). Health-Related Quality of Life and Service Barriers among Adults with Sickle Cell Disease in Saudi Arabia. Ethiop. J. Health Sci..

[12] Han H., Hensch L., Tubman V.N. (2021). Indications for transfusion in the management of sickle cell disease. Hematology.

[13] Tsitsikas D.A., Badle S., Hall R., Meenan J., Bello-Sanyaolu O., Orebayo F., Abukar J., Elmi M., Mulla A., Dave S. (2021). Automated Red Cell Exchange in the Management of Sickle Cell Disease. J. Clin. Med..

[14] Chou S.T., Alsawas M., Fasano R.M., Field J.J., Hendrickson J.E., Howard J., Kameka M., Kwiatkowski J.L., Pirenne F., Shi P.A. (2020). American Society of Hematology 2020 guidelines for sickle cell disease: Transfusion support. Blood Adv..

[15] Al Mozain N., Elobied Y., Al-Omran A., Aljaloud A., Omair A.B., Tuwaim R.B., Alkhalifah S., Altawil E.S., Abraham S., Salcedo L.R. (2023). Comparative study between chronic automated red blood cell exchange and manual exchange transfusion in patients with sickle cell disease: A single center experience from Saudi Arabia. Asian J. Transfus. Sci..

[16] Howard J. (2016). Sickle cell disease: When and how to transfuse. Hematol. Am. Soc. Hematol. Educ. Program.

[17] Kelly S. (2023). Logistics, risks, and benefits of automated red blood cell exchange for patients with sickle cell disease. Hematol. Am. Soc. Hematol. Educ. Program.

[18] Spectra Optia for Automatic Red Blood Cell Exchange in People with Sickle Cell Disease | Guidance | NICE n.d. https://www.nice.org.uk/guidance/mtg28.

[19] Tsitsikas D.A., Ekong A., Berg L., Hartzenberg J., Sirigireddy B., Lewis N., Solanki B., Amos R.J. (2017). A 5-year cost analysis of automated red cell exchange transfusion for the management of recurrent painful crises in adult patients with sickle cell disease. Transfus. Apher. Sci..

[20] Dedeken L., Lê P.Q., Rozen L., El Kenz H., Huybrechts S., Devalck C., Diallo S., Heijmans C., Ferster A. (2018). Automated RBC exchange compared to manual exchange transfusion for children with sickle cell disease is cost-effective and reduces iron overload. Transfusion.

[21] Willits I., Cole H., Jones R., Carter K., Arber M., Jenks M., Craig J., Sims A. (2017). Spectra Optia^®^ for Automated Red Blood Cell Exchange in Patients with Sickle Cell Disease: A NICE Medical Technology Guidance. Appl. Health Econ. Health Policy.

[22] Benmoussa K., Bernaudin F., Connes P., Héquet O., Joseph L., Beraud M., Bah A. (2024). Position paper on advancing sickle cell disease management in France by bridging the clinical practices and guidelines through expert insights. Transfus. Apher. Sci..

[23] Nasiri A., Alahmari A.D. (2023). The Quality of Life of Sickle Cell Disease Patients in Saudi Arabia 2023. Blood.

[24] Ezzat H.M., Al Zayed A.H., Al Saeed H.H., Al Darwish M., Albagshi M., Malhan H., Tarawah A., Al Manea A., Alhazmi I., Qari M. (2022). Sickle Cell Disease (SCD) Burden on Patients Treated with Hydroxyurea (HU): Results from the Real-World Assessment Survey for SCD in Saudi (ROARS). Blood.

[25] Bin Zuair A., Aldossari S., Alhumaidi R., Alrabiah M., Alshabanat A. (2023). The Burden of Sickle Cell Disease in Saudi Arabia: A Single-Institution Large Retrospective Study. Int. J. Gen. Med..

[26] Alotaibi M.M. (2017). Sickle cell disease in Saudi Arabia: A challenge or not. J. Epidemiol. Glob. Health.

[27] El-Mouzan M.I., Al-Salloum A.A., Al-Herbish A.S., Qurachi M.M., Al-Omar A.A. (2007). Regional variations in the prevalence of consanguinity in Saudi Arabia n.d. Saudi Med. J..

[28] Jastaniah W. (2011). Epidemiology of sickle cell disease in Saudi Arabia. Ann. Saudi Med..

[29] Alsultan A., Alabdulaali M.K., Griffin P.J., AlSuliman A.M., Ghabbour H.A., Sebastiani P., Albuali W.H., Al-Ali A.K., Chui D.H., Steinberg M.H. (2014). Sickle Cell Disease in Saudi Arabia: The Phenotype in Adults with the Arab-Indian Haplotype is not Benign. Br. J. Haematol..

[30] Adigwe O.P., Onoja S.O., Onavbavba G. (2023). A Critical Review of Sickle Cell Disease Burden and Challenges in Sub-Saharan Africa. J. Blood Med..

[31] Colombatti R., Birkegård C., Medici M. (2022). PB2215: Global Epidemiology of Sickle Cell Disease: A Systematic Literature Review. Hemasphere.

[32] Mangla A., Ehsan M., Agarwal N., Maruvada S. (2025). Sickle Cell Anemia.

[33] Busch M.P., Bloch E.M., Kleinman S. (2019). Prevention of transfusion-transmitted infections. Blood.

[34] Howard J. (2013). The role of blood transfusion in Sickle Cell Disease. ISBT Sci. Ser..

[35] Connelly-Smith L., Alquist C.R., Aqui N.A., Hofmann J.C., Klingel R., Onwuemene O.A., Patriquin C.J., Pham H.P., Sanchez A.P., Schneiderman J. (2023). Guidelines on the Use of Therapeutic Apheresis in Clinical Practice—Evidence-Based Approach from the Writing Committee of the American Society for Apheresis: The Ninth Special Issue. J. Clin. Apher..

[36] Sharma D., Kozanoğlu I., Ataga K.I., Benachi A., Büyükkurt S., Lanzkron S., Ozdogu H., Pancham S., Pecker L.H., Robinson S.E. (2024). Managing sickle cell disease and related complications in pregnancy: Results of an international Delphi panel. Blood Adv..

[37] Hulbert M.L., Scothorn D.J., Panepinto J.A., Scott J.P., Buchanan G.R., Sarnaik S., Fallon R., Chu J.Y., Wang W., Casella J.F. (2006). Exchange blood transfusion compared with simple transfusion for first overt stroke is associated with a lower risk of subsequent stroke: A retrospective cohort study of 137 children with sickle cell anemia. J. Pediatr..

[38] Kanter J., Liem R.I., Bernaudin F., Bolaños-Meade J., Fitzhugh C.D., Hankins J.S., Murad M.H., Panepinto J.A., Rondelli D., Shenoy S. (2021). American Society of Hematology 2021 guidelines for sickle cell disease: Stem cell transplantation. Blood Adv..

[39] Desai G., Dave K., Devare S., Desai S. (2024). Ethical and Clinical Considerations in the Use of Hydroxyurea in Pregnant Women with Sickle Cell Disease. Hemoglobin.

[40] Ballas S.K., McCarthy W.F., Guo N., DeCastro L., Bellevue R., Barton B.A., Waclawiw M.A., Investigators of the Multicenter Study of Hydroxyurea in Sickle Cell Anemia (2009). Exposure to hydroxyurea and pregnancy outcomes in patients with sickle cell anemia. J. Natl. Med. Assoc..

[41] Idris I.M., Burnett A.L., DeBaun M.R. (2022). Epidemiology and treatment of priapism in sickle cell disease. Hematol. Am. Soc. Hematol. Educ. Program.

[42] Ballas S.K., Lyon D. (2016). Safety and efficacy of blood exchange transfusion for priapism complicating sickle cell disease. J. Clin. Apher..

[43] Padmanabhan A., Connelly-Smith L., Aqui N., Balogun R.A., Klingel R., Meyer E., Pham H.P., Schneiderman J., Witt V., Wu Y. (2019). Guidelines on the Use of Therapeutic Apheresis in Clinical Practice—Evidence-Based Approach from the Writing Committee of the American Society for Apheresis: The Eighth Special Issue. J. Clin. Apher..

[44] Dillman J.R., Ellis J.H., Cohan R.H., Caoili E.M., Hussain H.K., Campbell A.D., Strouse P.J. (2011). Safety of gadolinium-based contrast material in sickle cell disease. J. Magn. Reson. Imaging.

[45] Tsitsikas D.A., Mihalca D., Hibbs S., Freeman T., Bello-Sanyaolu O., Orebayo F., Lewis N., Green L. (2022). Therapeutic plasma exchange in the management of acute complications of sickle cell disease: A single centre experience. Transfus. Apher. Sci..

[46] Webb C.B., Yates S.G., Sarode R., Kim J. (2023). Plasma exchange-A useful adjunct therapy to red cell exchange in patients with sickle cell disease and multiorgan dysfunction. Transfusion.

